# Correction: ^19^F multiple-quantum coherence NMR spectroscopy for probing protein–ligand interactions

**DOI:** 10.1039/c9ra90004g

**Published:** 2019-01-25

**Authors:** Anna Zawadzka-Kazimierczuk, Mate Somlyay, Hanspeter Kaehlig, George Iakobson, Petr Beier, Robert Konrat

**Affiliations:** Department of Structural and Computational Biology, Max F. Perutz Laboratories, University of Vienna Vienna Biocenter Campus 5 A-1030 Vienna Austria; Biological and Chemical Research Centre, Faculty of Chemistry, University of Warsaw Żwirki i Wigury 101 02-089 Warsaw Poland; Institute of Organic Chemistry, University of Vienna Währinger Strasse 38 A-1090 Vienna Austria; Institute of Organic Chemistry and Biochemistry of the Czech Academy of Sciences Flemingovo nam. 2 160 00 Prague Czech Republic anzaw@chem.uw.edu.pl Robert.Konrat@univie.ac.at

## Abstract

Correction for ‘^19^F multiple-quantum coherence NMR spectroscopy for probing protein–ligand interactions’ by Anna Zawadzka-Kazimierczuk *et al.*, *RSC Adv.*, 2018, **8**, 40687–40692.

In the original manuscript, Fig. 1 contained an error, for which two pulsed field gradients in the NMR pulse sequences depicted should not be present. The correct figure and caption are as follows.

**Fig. 1:**
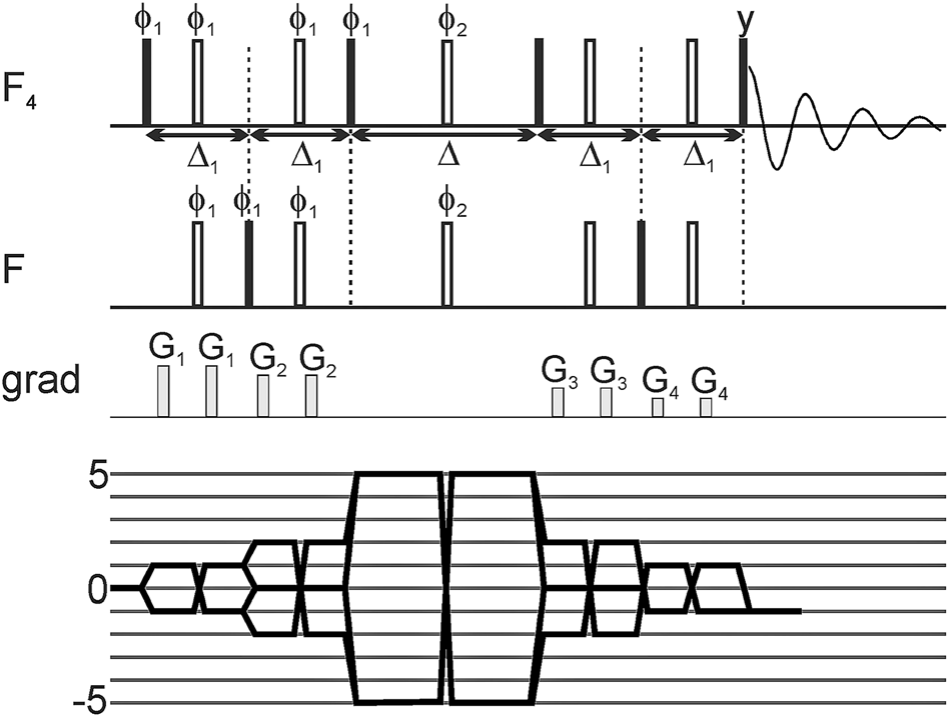
Scheme of the 5Q pulse sequence for *T*_2_ measurements of the SF_5_ system, together with coherence transfer pathway. All pulses were selective shaped pulses. The pulses acting on one type of fluorine nuclei (F or F_4_ group) were sinc-shaped; their length was set to 250 μs and offset was set on the frequency of the group. The pulses applied simultaneously on F and F_4_ groups were cosine-modulated sinc-shaped pulses; their length was 250 μs and offset was set in the middle between the two frequencies. The Δ_1_ delay was set to 6.28 ms and the relaxation delay Δ was incremented. The pulse phases were set to *x*, unless shown explicitly. On the phase *ϕ*_1_ a 10-step phase cycle was performed to select the coherence of ±5 order during the multiple-quantum period. Additionally, on the phase *ϕ*_2_ a 4-step phase cycle was performed.

The Royal Society of Chemistry apologises for these errors and any consequent inconvenience to authors and readers.

## Supplementary Material

